# Argon plasma-activated liquid inactivates *Helicobacter pylori* and resistant hospital pathogens through acidification and reactive species

**DOI:** 10.3389/fmicb.2026.1771100

**Published:** 2026-02-16

**Authors:** Leander Heisterberg, Ulrich Biber, Alexander Neugebauer, Jan Liese, Christopher C. Thompson, Markus Enderle, Sebastian Grashorn

**Affiliations:** 1 Erbe Elektromedizin GmbH, Tübingen, Germany; 2 Division of Gastroenterology, Hepatology and Endoscopy, Brigham and Women’s Hospital, Boston, MA, United States; 3 Department of Medicine, Harvard Medical School, Boston, MA, United States; 4 Tübingen University Hospital, Institute of Medical Microbiology and Hygiene, Tübingen, Germany

**Keywords:** cold atmospheric plasma, *Helicobacter pylori*, microbial biotechnology, multidrug-resistant bacteria, novel antimicrobials, plasma activated liquid

## Abstract

**Background:**

The rise of antibiotic-resistant bacteria is a major public health concern. Physical plasma can generate reactive oxygen and nitrogen species (RONS) with antimicrobial activity. Plasma-activated liquid (PAL) can be used as a carrier for RONS. This study investigated the antibacterial effects of PAL against clinically relevant Gram-negative (*Escherichia coli*, multidrug-resistant (MDR) *Escherichia coli*, *Pseudomonas aeruginosa*, *Acinetobacter baumannii*, *Enterobacter cloacae*, *Klebsiella pneumoniae*, and *Helicobacter pylori*) and Gram-positive (*Staphylococcus aureus* and *Enterococcus faecium*) bacteria.

**Methods and results:**

A commercial argon plasma electrosurgical source was used to produce PAL from 0.9% NaCl solution. PAL-NaCl showed strong bactericidal effects: MDR *E. coli* was completely eradicated (>6 log_10_ reduction) within 60 min. *H. pylori* was reduced by ~4.5 log_10_ within 15 min and completely eradicated (>5 log_10_) within 60 min of exposure. Gram-negative bacteria were highly susceptible with a mean reduction of ~6.3 log_10_, whereas Gram-positive bacteria showed lower susceptibility with a mean reduction of ~2.6 log_10_. Antibacterial activity was associated with acidic pH and influenced by carrier solution chemistry, consistent with a contribution of short-lived secondary reactive nitrogen species. The scavenger 5,10,15,20-tetrakis(4-sulfonatophenyl)porphyrinato iron(III) chloride (FeTPPS), targeting peroxynitrite (ONOO^−^), partially inhibited the antibacterial effect, supporting its mechanistic importance. Buffered solutions (higher pH) showed minimal antibacterial activity despite higher absolute RONS concentrations which underlines the importance of the acidic environment.

**Conclusion:**

PAL generated with an argon plasma electrosurgical source exhibits potent antibacterial activity, driven by low pH and RONS dynamics. PAL effectively inactivates MDR pathogens and other clinically relevant pathogens, including *H. pylori*. The stomach’s acidic environment may enhance PAL activity by maintaining the low-pH conditions required for pH-dependent reactive nitrogen chemistry associated with antibacterial efficacy.

## 1 Introduction

The rise of antibiotic-resistant pathogens is an increasingly critical global health issue. In clinical settings, pathogens such as *Helicobacter pylori* (*H. pylori*) and the so-called ESKAPE pathogens (*Enterococcus faecium*, *Staphylococcus aureus*, *Klebsiella pneumoniae*, *Acinetobacter baumannii*, *Pseudomonas aeruginosa*, and *Enterobacter* species) pose significant challenges due to rising resistance profiles (Salam et al., 2023; Rotondo et al., 2025). For *H. pylori*, the eradication success rate is steadily decreasing with most studies reporting rates ≤80%, despite aggressive therapies using combinations of proton pump inhibitors (PPIs) and antibiotics (Malfertheiner et al., 2012). With the increasing emergence of multidrug-resistant (MDR) bacterial strains, there is a need to explore alternative approaches with mechanisms of action other than antibiotics (Argueta et al., 2021).

Low-thermal plasma (LTP) has emerged as a promising candidate that exhibits potent antimicrobial activities (Das et al., 2022; Koga-Ito et al., 2023). Here, LTP is used in an application-oriented sense and refers to plasma operation in which thermal effects remain below the threshold for visible tissue coagulation. In plasma-physics terms, this corresponds to non-equilibrium (non-thermal) atmospheric plasmas, often termed cold atmospheric plasma (CAP), rather than thermal plasmas in (near) thermal equilibrium (Adamovich et al., 2022). Clinically, LTP treatments have been used in wound management, demonstrating successful reduction of microbial burden and improved wound healing outcomes (Amini et al., 2020; Bekeschus et al., 2021; Talukdar et al., 2025). These are driven by UV radiation, charged particles, electrons, ions, mild heat and reactive oxygen and nitrogen species (RONS), including hydrogen peroxide (H_2_O_2_), nitrite (NO_2_^−^), nitrate (NO_3_^−^), and peroxynitrite (ONOO^−^) (Kaushik et al., 2018). Plasma-based technologies have demonstrated broad-spectrum efficacy against a variety of pathogens, including both Gram-positive (e.g., *Staphylococcus aureus*) and Gram-negative bacteria (e.g., *Escherichia coli*, *Pseudomonas aeruginosa*), as well as fungi such as *Candida albicans* and *Aspergillus* spp. (Kim and Kim, 2021).

Plasma activated liquids (PALs) are created by applying physical plasma to a liquid. PALs are liquids in which plasma-generated reactive species are dissolved and can be delivered to less accessible sites. The antimicrobial effect of PAL has been shown in numerous studies (Liu et al., 2010; Traylor et al., 2011; Wang et al., 2020; Zhao et al., 2020). This process is already used commercially to some extent for non-medical applications, e.g., the use of PAL in agriculture for antibacterial and antifungal effects (Puač and Škoro, 2025). Although there has been a lot of preclinical biomedical research, standardized and clinically scalable PAL generation and workflows remain limited (Laroussi et al., 2022; Milhan et al., 2022).

Various plasma sources have been used to generate PAL, including dielectric barrier discharge (DBD), plasma jets, and transient spark discharge devices (Weltmann et al., 2010; Gohain and Biswas, 2025). Currently, there are only a few medically approved plasma devices on the market, most notably the kINPen^®^ MED (neoplas med GmbH, Greifswald, Germany), which is primarily indicated for wound healing applications but has also been used experimentally to generate PAL (Reuter et al., 2018; Clemen et al., 2023).

More recently, argon plasma coagulation (APC) devices have also been studied for their LTP effects (Weiss et al., 2019; Martinet et al., 2025). These are approved medical devices that have been on the market for over 30 years (Grund et al., 1997; Grund et al., 1999). By minimizing the effect setting (e.g., electrical power of 1 W or even less), they can be operated in a low-thermal mode, which limits or even omits the thermal effect and coagulation of tissue (Marzi et al., 2022; Weiss et al., 2023). It has been shown that these low-thermal modes generate RONS in an electrical power level dependent manner, with more RONS species being generated at higher power settings and fewer RONS being generated at lower power settings (Martinet et al., 2025). While higher APC power settings increase the generation of RONS, they are not suitable for direct low-thermal plasma application due to increasing thermal effects and tissue coagulation (Zenker, 2008; Martinet et al., 2025). In contrast, when plasma is applied to liquids, thermal effects are decoupled from RONS generation, as the liquid acts as a heat sink due to the high heat capacity of aqueous solutions and allows the production of PAL with high RONS concentrations (Bruggeman et al., 2016; Weiss et al., 2019).

Previous PAL studies have shown that antibacterial activity depends strongly on liquid composition, acidification, and reactive species chemistry, including synergistic interactions between hydrogen peroxide and nitrite under acidic conditions (Oehmigen et al., 2010; Wang and Salvi, 2021; Laroussi et al., 2022). However, reported efficacy varies widely, in part because PAL is frequently generated using laboratory plasma sources with heterogeneous operating conditions, limiting comparability and reproducibility across studies.

A translational barrier is the limited availability of clinically certified and scalable PAL generation platforms. Electrosurgical APC systems are widely available in hospitals and provide adjustable energy delivery, but APC-generated PAL has not been systematically evaluated against clinically relevant organisms such as *H. pylori* and MDR hospital isolates.

This study investigates the antibacterial effect and gains first insights into mechanisms of action of a commercially available plasma source—APC—on clinically relevant bacterial strains commonly found in hospital settings. The primary focus is on *H. pylori*, whose susceptibility to PAL has not been systematically evaluated. *H. pylori* is a prevalent gastric pathogen that causes chronic gastritis and peptic ulcer disease and is a major risk factor for gastric cancer, with eradication rates declining due to increasing antibiotic resistance (Hooi et al., 2017; Liou et al., 2020). In addition, we investigated MDR *E. coli* strain and ESKAPE pathogens. Physicochemical properties of PAL were analyzed, and antibacterial effects were assessed under defined conditions. First mechanistic insights were obtained using chemical controls and selected radical scavengers for selected RONS.

## 2 Material & methods

### 2.1 PAL generation setup

**Figure 1:**
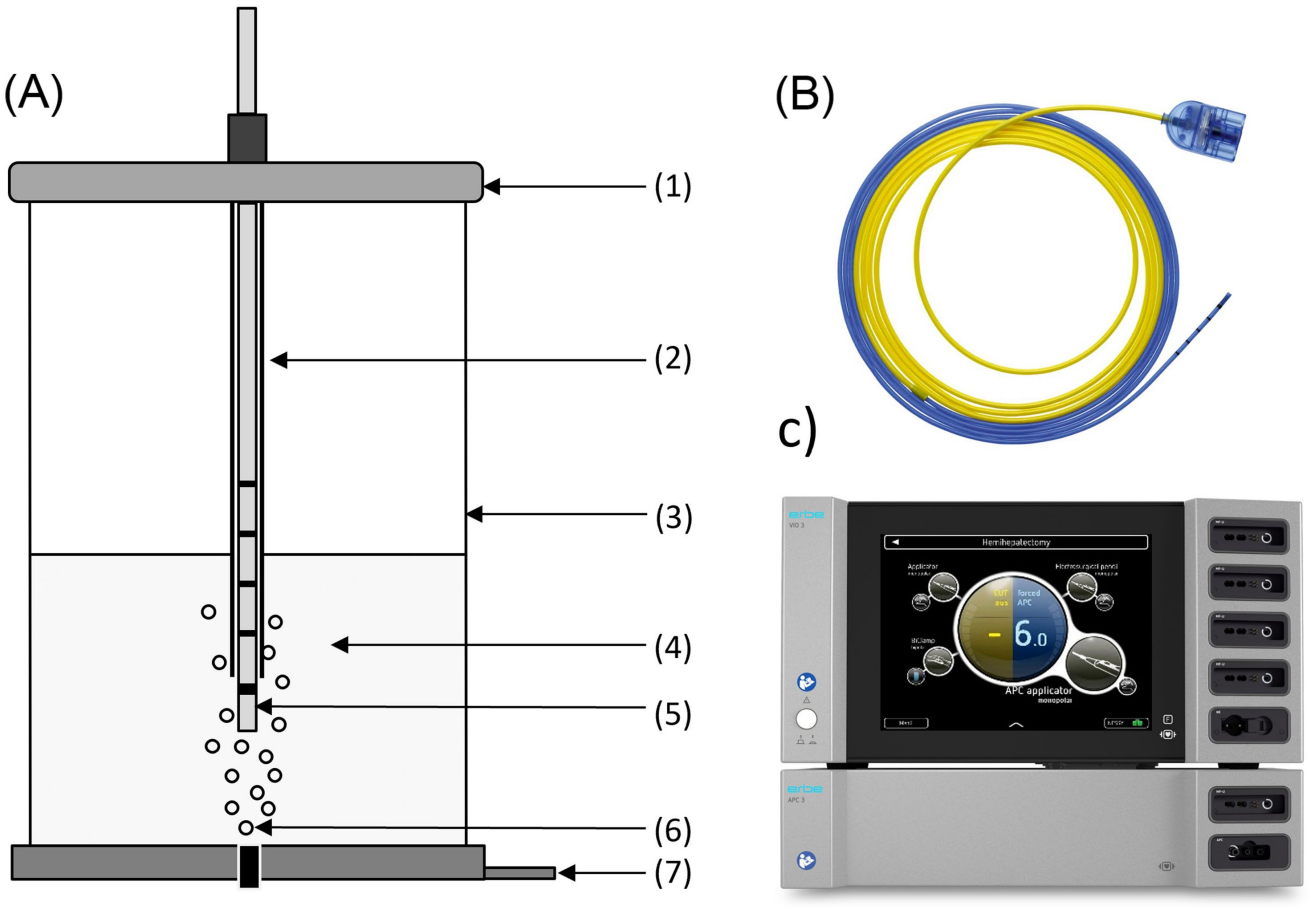
PAL generation setup. **(A)** PAL reactor vessel with (1) lid, (2) sleeve, (3) vessel, (4) liquid, (5) FiAPC probe, (6) gas outlet with bubbling, (7) neutral electrode. **(B)** Close-up of the flexible FiAPC probe. **(C)** The electrosurgical generator (VIO^®^3) equipped with the argon plasma module (APC3).

PAL was generated using a medical argon plasma system (VIO^®^3 with APC3 module, Erbe Elektromedizin GmbH, Tübingen, Germany) connected to a flexible Filter integrated Argon Plasma Coagulation (FiAPC) probe. Plasma was applied using the VIO3 mode forcedAPC effect setting 8.0 with an argon flow rate of 0.3 L/min. The PAL reactor vessel supplied air bubbles from the bottom at an airflow of 0.5 L/min and the probe tip (immersed into the liquid) was held at a fixed 4 mm distance from a stainless-steel neutral electrode (Figure 1). For each run, 50 mL of either 0.9% NaCl (NaCl, B. Braun, Melsungen, Germany) or Ringer’s lactate (Ri-Lac, B. Braun, Melsungen, Germany) were used as carrier solutions.

### 2.2 Physicochemical properties of PAL used in antibacterial assays

PAL was generated using plasma activation times of 150 s (low-dose) and 225 s (standard). The 225 s condition was selected as the standard because it yielded consistent PAL chemistry and strong antibacterial effects in our setup, while 150 s was included to assess dose dependence. Unless stated otherwise, antibacterial experiments were performed using PAL generated with an activation time of 225 s (PAL-NaCl).

PAL was characterized by measuring pH and the concentrations of H₂O₂ and NO₂^−^ immediately after plasma activation. The pH was measured using a calibrated pH meter (FiveEasy F20, Mettler-Toledo, Gießen, Germany).

Concentrations of H_2_O_2_ and NO_2_^−^ were measured photometrically using a Spectroquant^®^ Prove 100 system (Merck/Supelco, Darmstadt, Germany). This method was previously described and validated for PAL measurements (Veronico et al., 2021). Samples were appropriately diluted to fit within the assay range, followed by reaction with specific reagents for H_2_O_2_ (Supelco Hydrogen Peroxide Cell Test, Merck, Darmstadt, Germany) and NO_2_^−^ (Supelco Nitrite Test, Merck, Darmstadt, Germany). Reactions were allowed to proceed for 10 min before photometric analysis. All measurements were performed in independent triplicates.

PAL-NaCl showed a pH of 2.94 ± 0.03, with hydrogen peroxide concentrations of 670 ± 17 μmol/L and nitrite concentrations of 148 ± 35 μmol/L. In contrast, PAL generated from Ringer’s lactate at 225 s remained weakly acidic (pH 5.03 ± 0.05) with substantially higher concentrations of hydrogen peroxide (1,287 ± 111 μmol/L) and nitrite (1,269 ± 96 μmol/L). PAL generated from 0.9% NaCl with an activation time of 150 s (low-dose PAL) exhibited a pH of 3.13 ± 0.02 and contained hydrogen peroxide and nitrite at concentrations of 616 ± 41 μmol/L and 473 ± 7 μmol/L, respectively.

### 2.3 Bacterial strains, culture preparation and PAL exposure

All microbiological experiments were conducted at the Institute for Medical Microbiology and Hygiene, University Hospital Tübingen, Germany. The following bacterial strains were tested in the sensitivity assay: Gram-positive *Staphylococcus aureus* ATCC 6538 (*S. aureus*), *Enterococcus faecium* ATCC 6057 (*E. faecium*); and Gram-negative *Escherichia coli* NCTC 10538 (*E. coli*), *Pseudomonas aeruginosa* ATCC15442 (*P. aeruginosa*), *Acinetobacter baumannii* ATCC 19606 (*A. baumannii*), *Enterobacter cloacae* DSM BAA-143 (*E. cloacae*), *Klebsiella pneumoniae* ATCC 700603 (*K. pneumoniae*), *Helicobacter pylori* SK225 (*H. pylori*). In addition, a multidrug-resistant clinical isolate [*Escherichia coli* strain *4MRGN E. coli* (MDR), internal freezing number VC8874] was tested.

For each experiment, fresh cultures were prepared from frozen stocks. Bacterial strains were grown on Columbia blood agar with sheep blood (Ref. PB50339A, Thermo Fisher Scientific, Waltham, United States), while *H. pylori* was cultured on selective Pylori agar (Ref. 413193, bioMérieux, Nürtingen, Germany). Plates were incubated at 37 °C for 24 h (all strains except *H. pylori*) or 72 h (*H. pylori*). For each experiment, bacterial colonies were suspended in 0.9% NaCl solution and adjusted to a McFarland standard of 0.5. The actual bacterial concentration of each inoculum was verified for every experimental batch by colony-forming unit (CFU) enumeration using serial dilution and plating.

For PAL exposure, 9 mL of freshly generated PAL (temperature immediately after activation: 55.5 ± 2.5 °C) was transferred into sterile reaction tubes and cooled in an ice bath to <37 °C before mixing with 1 mL bacterial suspensions. This resulted in a starting concentration of approximately 1.5 × 10^7^ CFU/mL. Control samples were prepared using 0.9% NaCl at room temperature instead of PAL. The first sampling time point (0 min) corresponded to approximately 15–30 s of contact time, accounting for cooling, mixing, and handling. Samples were collected at 0, 15, 60, and 180 min, serially diluted in 0.9% NaCl (up to 10^−6^), and 100 μL of each dilution was plated in duplicate on the corresponding agar medium. Plates were incubated at 37 °C for 24 h (or 72 h for *H. pylori*), and viable bacteria were quantified by CFU counting.

All experiments were performed *in vitro*. Antibacterial activity of PAL was assessed using a suspension assay against the strain panel listed in Section 2.3 above. Subsequent experiments (controls, time-course, and scavenger assays) were performed on selected representative strains as specified in the respective Results subsections.

Control conditions included untreated 0.9% NaCl, PAL generated from Ringer’s lactate solution, acidified 0.9% NaCl (pH 2.8, adjusted with hydrochloric acid), and acidified 0.9% NaCl supplemented with hydrogen peroxide (final concentration 25 mg/L). The experimental procedure was identical for all conditions.

Each experiment was performed in at least three independent biological replicates. Within each biological replicate, samples were plated in technical duplicate, and colony counts were averaged prior to statistical analysis.

### 2.4 Co-exposure with RONS scavengers

To assess the contribution of selected short-lived reactive oxygen and nitrogen species to the antibacterial activity of PAL, chemical scavengers were used to selectively target specific reactive pathways. The following scavengers were applied based on established literature: 5,10,15,20-tetrakis(4-sulfonatophenyl)porphyrinato iron(III) chloride (FeTPPS) for peroxynitrite, L-histidine for singlet oxygen, D-mannitol for hydroxyl radicals, and taurine for hypochlorous acid (Bauer et al., 2019; Ma et al., 2020; Kang et al., 2022; Xu et al., 2022).

All scavengers were obtained from Sigma-Aldrich (Merck, Darmstadt, Germany). Stock solutions were freshly prepared in distilled water and added to PAL to achieve final concentrations of 40 μM FeTPPS, 2 mM L-histidine, 20 mM D-mannitol, or 50 mM taurine. To avoid pH-related confounding effects, the pH of the L-histidine stock solution was adjusted to 2.8 ± 0.2 prior to use.

For each experiment, freshly generated PAL (activation time 225 s) was distributed into reaction tubes containing either no additive (PAL control) or one of the scavengers. Immediately after mixing by vortexing, pH as well as H_2_O_2_ and NO_2_^−^ levels were verified using QUANTOFIX^®^ test strips and reflectometric analysis (MACHEREY-NAGEL, Düren, Germany). PAL–scavenger mixtures were then used directly for antibacterial assays as described above.

### 2.5 Statistical analysis

All experiments were performed using at least three independent biological replicates (*n* ≥ 3). Log₁₀ reduction values were calculated for each strain and condition. Descriptive statistics, including mean and standard deviation, were calculated. The normal distribution of data sets was evaluated using a Shapiro–Wilk normality test. Since the data sets were normally distributed, differences between data sets were assessed using Student’s *t*-tests. One sample *t*-tests against a hypothetical value of zero were used to assess if a log_10_ reduction was significant at all. *p*-values <0.05 were considered statistically significant. All analyses were performed using GraphPad PRISM, GraphPad, Boston, United States.

## 3 Results

### 3.1 Antibacterial susceptibility testing

**Figure 2:**
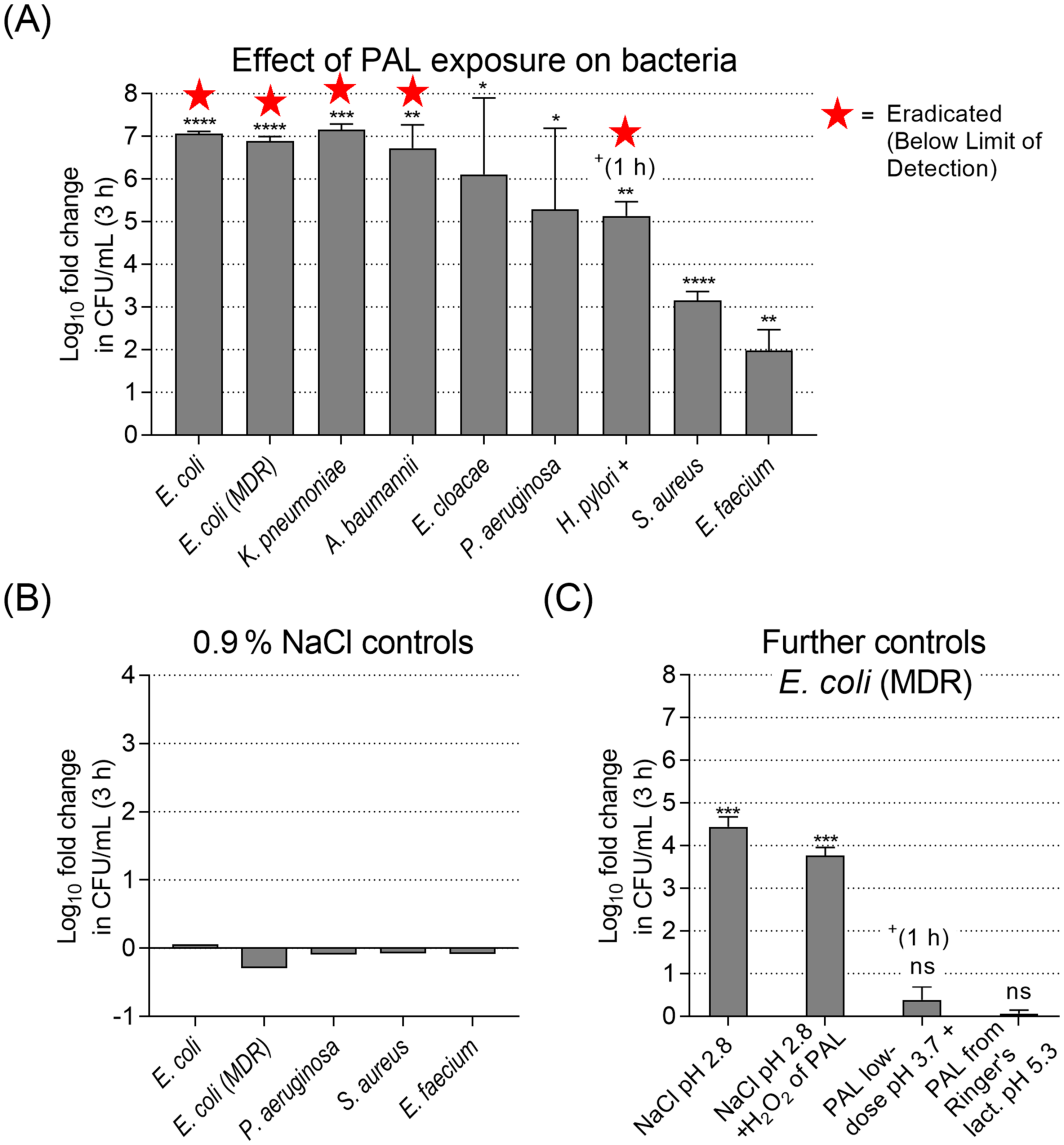
Bacterial suspension assay of different strains, mean log_10_ fold reduction across independent experiments after 3 h exposure: **(A)** with PAL (*n* = 3 independent experiments). Star symbols indicate complete eradication (reduction to below the limit of detection, <10 CFU/mL). PAL exposure resulted in antibacterial effects ranging from complete eradication (≥6-log reduction) for both *E. coli* strains, *K. pneumoniae* and *A. baumannii*, to a strong reduction for *E. faecium* (~2-log reduction). **(B)** Stability controls showing the log_10_ fold change of bacteria in untreated 0.9% NaCl between 0 h and 3 h. Values near zero indicate no significant growth or death during incubation. **(C)** Further mechanistic controls for *E. coli* (MDR) (*n* = 3). Four agar plates of a dilution series were evaluated per biological replicate (*n*). Error bars show SD. ^*^p < 0.05, ^**^p < 0.01, ^***^p < 0.001, and ^****^p < 0.0001, ns, not significant (one-sample *t*-test vs. 0).

The antibacterial activity of PAL was evaluated against a panel of Gram-negative and Gram-positive bacterial strains (Figure 2) using a suspension assay with a total exposure time of 3 h (1 h for *H. pylori* due to rapid inactivation). Bacterial viability after PAL exposure was quantified as log_10_ reduction compared to the respective 0.9% NaCl-treated control samples.

PAL exposure (Figure 2A) resulted in potent bactericidal effects for all tested Gram-negative strains. Complete eradication or high susceptibility (≥5 log_10_ reduction) was observed for *E. coli*, *E. coli* (MDR), *K. pneumoniae*, *A. baumannii*, *E. cloacae*, *P. aeruginosa*, and *H. pylori*. These reductions were statistically significant for the majority of strains (one-sample *t*-tests: *p* < 0.0001 to *p* < 0.05), with the exception of *P. aeruginosa* (*p* = 0.19) due to higher variance between replicates. In contrast, Gram-positive strains showed lower susceptibility. *S. aureus* exhibited a reduction of ~3.2 log_10_ (*p* < 0.001) and *E. faecium* a reduction of ~2.0 log_10_ (*p* = 0.076). No significant difference in susceptibility was observed between the non-resistant and the MDR *E. coli* strains (*p* = 0.11).

We performed a NaCl-only control series (Figure 2B) with several bacterial strains with 3 h exposure before plating on agar. No marked changes in bacterial concentrations were detected.

We further investigated the influencing factors driving this toxicity using *E. coli* (MDR) controls (Figure 2C). Antibacterial effects were observed in these tests, but with considerable differences: acidified 0.9% NaCl (pH 2.8) achieved a significant ~4.4 log_10_ reduction (*p* < 0.001). With 0.9% NaCl adjusted to a pH of 2.8 and the addition of H_2_O_2_ corresponding to the PAL concentration, a ~ 3.8 log_10_ reduction was obtained (*p* < 0.001). Conversely, low-dose PAL (150 s activation, 1 h exposure) and PAL produced from Ringer’s lactate solution (pH 5.3) resulted in non-significant reductions of <1 log_10_ (*p* = 0.16 and *p* = 0.31, respectively).

### 3.2 Time course of antibacterial effect

**Figure 3:**
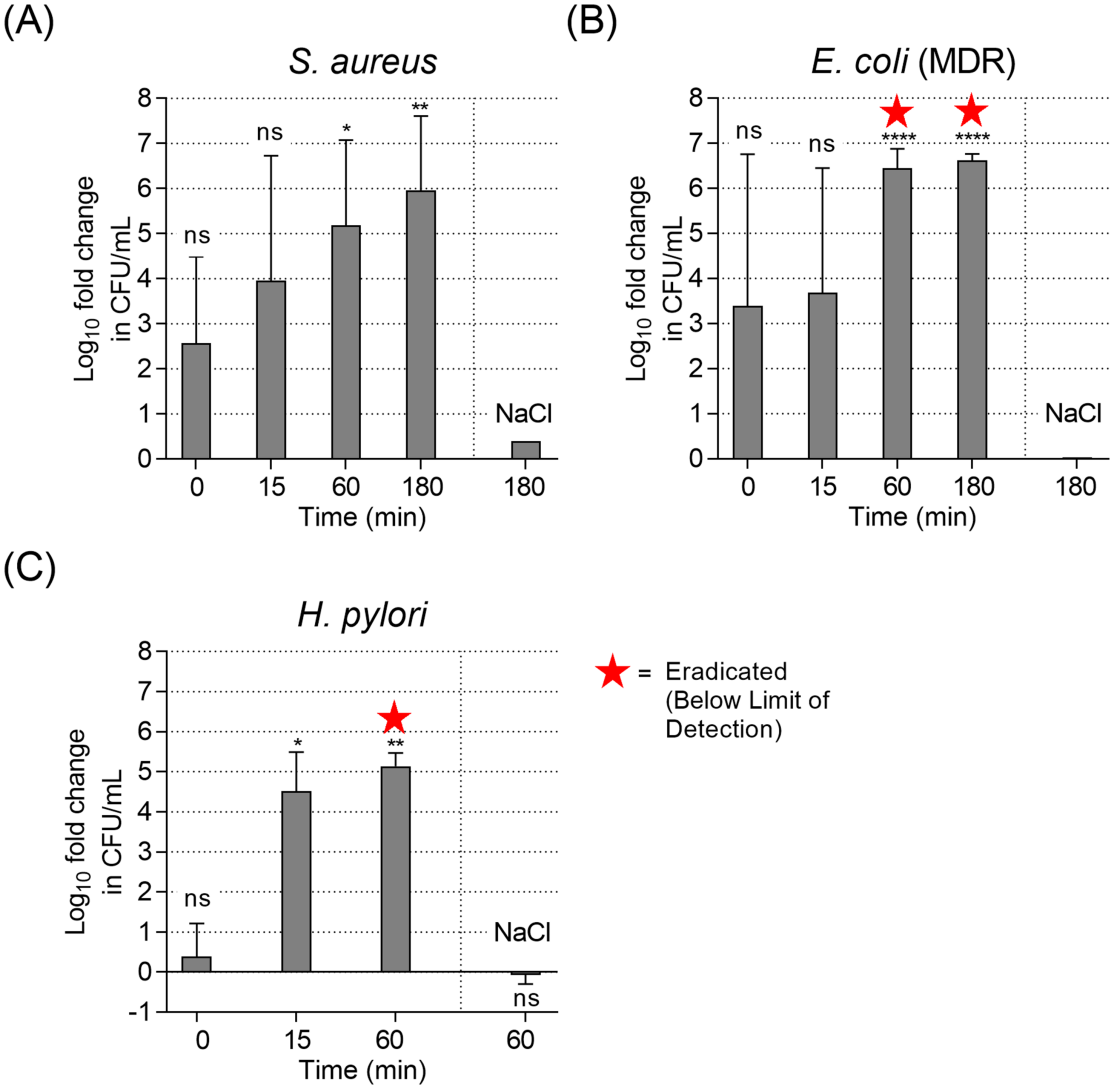
Bacterial suspension assay of different strains with various exposure times, mean log_10_ fold reduction across independent experiments: **(A)** *S. aureus* (*n* = 4 replicates), **(B)** *E. coli* (MDR) (*n* = 3–4 replicates), and **(C)** *H. pylori* (*n* = 3): with PAL exposure and with 0.9% NaCl control treatment at the end of a single experiment (60 or 180 min). Error bars show SD. ^*^p < 0.05, ^**^p < 0.01, ^***^p < 0.001, and ^****^p < 0.0001, ns, not significant (one-sample *t*-test vs. 0).

The antibacterial time-dependency of PAL were evaluated for *S. aureus*, *E. coli* (MDR) and *H. pylori* with exposure times of 0, 15, 60 and 180 min (Figure 3). For *S. aureus* (Figure 3A), PAL exposure resulted in a progressive, time-dependent decrease in bacterial viability. Immediately after exposure (0 min, <30 s contact time), a reduction of ~2.6 log_10_ was observed, though this initial effect was not yet statistically significant (*p* = 0.074). The efficacy increased to a ~ 4.0 log_10_ reduction after 15 min (*p* = 0.065) and reached a significant ~5.2 log_10_ reduction after 60 min (*p* = 0.012). In the case of *E. coli* (MDR) (Figure 3B), PAL exposure led to an immediate but variable drop in bacterial density. At 0 min, a mean reduction of ~3.4 log_10_ was recorded; however, due to high variability between replicates, this early reduction did not reach statistical significance (*p* = 0.22). A similar effect was observed at 15 min (*p* = 0.15). At 60 and 180 min a complete eradication (>6 log_10_ reduction) was achieved (*p* < 0.0001). *H. pylori* (Figure 3C) exhibited the most rapid susceptibility. After 15 min of exposure, a significant ~4.5 log_10_ reduction to 24 ± 10 CFU/mL was observed (*p* = 0.015). Complete eradication (>5 log_10_ reduction) was achieved after 60 min, with no viable colonies detected in any treated samples (*p* < 0.01).

### 3.3 PAL modified by radical scavengers

**Figure 4:**
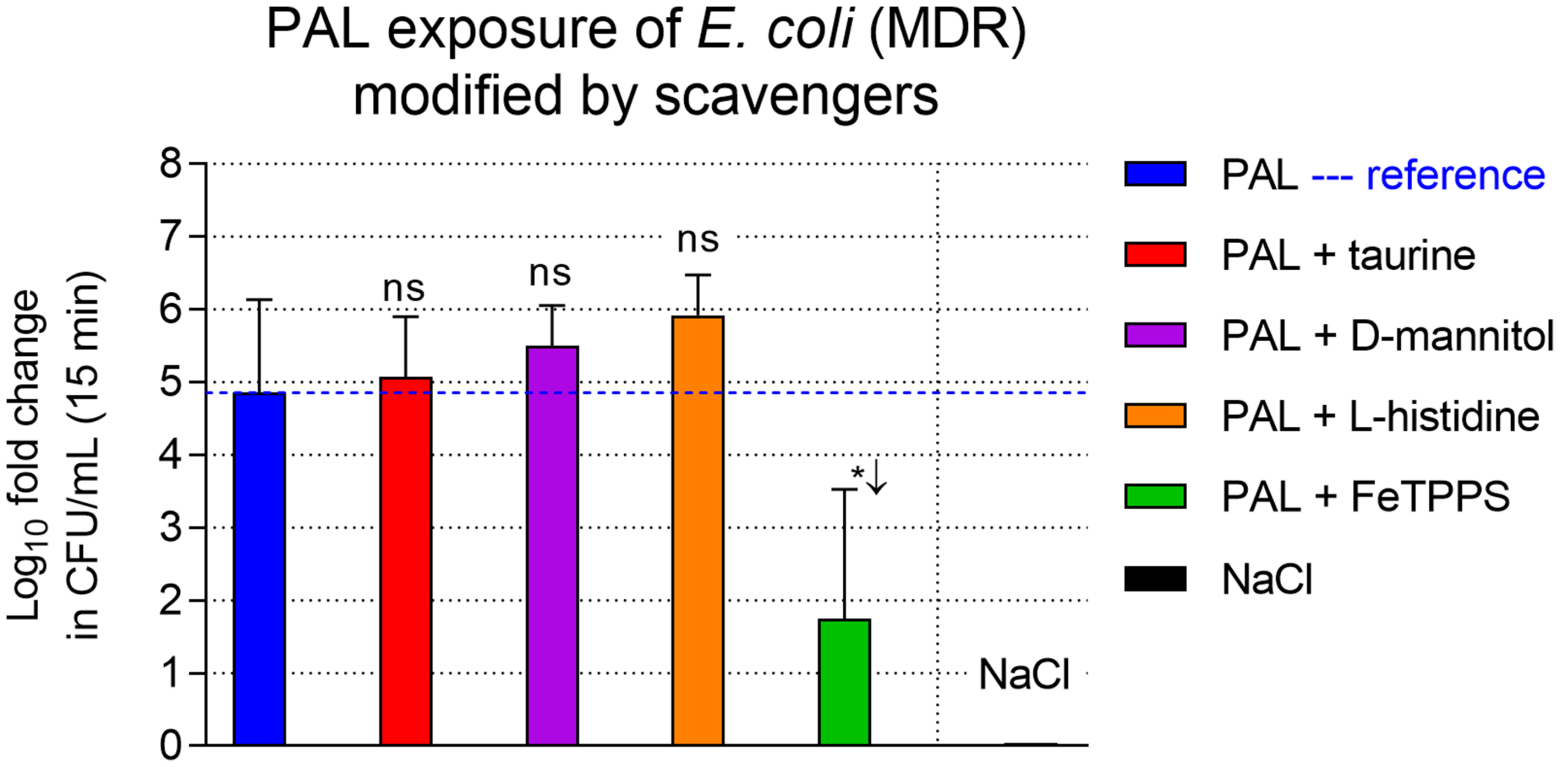
Bacterial suspension assay of *E. coli* (MDR), mean log_10_ fold reduction across independent experiments after 15 min exposure with standard PAL (blue) and with various PALs modified by radical scavengers (colored bars) and with 0.9% NaCl (black). The PAL-NaCl exposure served as reference (log_10_ fold reduction: 4.85), marked as blue dashed line. FeTPPS, that catalytically isomerizes peroxynitrite to nitrate, reducing efficacy to <1.8 log_10_, whereas the other scavengers did not. Error bars show SD. Pairwise *t*-tests between PAL-NaCl and the modified PAL groups are graphed: ^*^p < 0.05, ns, not significant.

To identify the specific reactive species driving bactericidal activity, we modified the PAL-NaCl by adding selective radical scavengers (Figure 4). This series was conducted using *E. coli* (MDR). As a reference, PAL-NaCl alone (15 min exposure) reduced bacterial viability to 1740 ± 241 CFU/mL, corresponding to a significant ~4.9 log_10_ reduction (*p* < 0.01). The addition of taurine (scavenger for HOCl), D-mannitol (hydroxyl radicals), or L-histidine (singlet oxygen) adjusted to pH 2.8 did not inhibit antimicrobial efficacy. In all three cases, the reduction was comparable to or greater than the PAL-NaCl reference (5 log_10_ reduction; *p* = 0.80, *p* = 0.45 and *p* = 0.23, respectively). In contrast, the addition of FeTPPS, a catalyst for the decomposition of peroxynitrite (ONOO^−^), significantly attenuated the bactericidal effect (*p* = 0.028 vs. PAL-NaCl). In the presence of FeTPPS, the bacterial density remained high at 881 × 10^3^ ± 71.9 × 10^3^ CFU/mL (~1.8 log_10_ reduction, *p* = 0.23), whereas no reduction was observed in the NaCl control.

## 4 Discussion

PALs have been widely investigated as non-antibiotic antimicrobial approaches, but reported effects vary substantially across studies due to differences in plasma sources, activation conditions, and liquid composition (Kim and Kim, 2021; Koga-Ito et al., 2023; von Woedtke et al., 2025). A practical limitation is that many PAL studies rely on laboratory plasma devices that are not standardized or clinically available, complicating comparison and reproducibility (Montalbetti et al., 2025). In this study, we evaluated the antibacterial activity of PAL generated by a clinically certified electrosurgical APC device. We demonstrated rapid and potent antibacterial effects of APC-derived PAL against multiple Gram-negative pathogens, including *H. pylori* and *E. coli* (MDR). Gram-positive bacteria exhibited significant but comparatively lower susceptibility. Our results emphasize the critical role of low pH in achieving effective antibacterial activity. RONS dynamics, particularly the rapid consumption of nitrite under acidic conditions leading to the formation of peroxynitrous acid (ONOOH) and its conjugate base peroxynitrite (ONOO^−^) as well as peroxynitric acid (O_2_NOOH), appear central to this bactericidal action. Importantly, buffered solutions maintaining higher pH levels were ineffective.

### 4.1 RONS dynamics and pH dependency impact antibacterial efficacy

Our results confirm that plasma activation of liquids generates H_2_O_2_ and NO_2_^−^, and that their antibacterial efficacy strongly depends on carrier solution chemistry and resulting pH. PAL generated from 0.9% NaCl reached acidic pH values below 3, whereas Ringer’s lactate remained weakly acidic (≈pH 5) due to its buffering capacity. Antibacterial activity tracked with acidity rather than with the absolute concentrations of long-lived species: despite higher H_2_O_2_/NO_2_^−^ levels, Ringer’s lactate-derived PAL showed minimal killing, while acidified conditions were bactericidal. Similar pH-dependent effects of PAL have been reported previously (Ikawa et al., 2010; Oehmigen et al., 2010).

Under acidic conditions, interactions between H_2_O_2_ and NO_2_^−^ can give rise to secondary reactive nitrogen species such as peroxynitrous acid and peroxynitrite, which have been implicated in antimicrobial activity (Wang and Salvi, 2021; Zhao et al., 2021; Rotondo et al., 2025). Consistent with this, peroxynitrite scavenging partially attenuated antibacterial activity, supporting a contribution of short-lived secondary chemistry rather than long-lived RONS alone. Practically, this strong pH dependence implies that buffering capacity and handling time are critical determinants of reproducibility, as modest pH shifts can suppress PAL activity.

Differences in reported antibacterial efficacy of PAL across the literature may, in part, be explained by methodological variations. In studies demonstrating bacterial inactivation at higher bulk pH, microorganisms were frequently exposed directly to plasma within the liquid, potentially generating localized acidic microenvironments and introducing additional antimicrobial factors such as ultraviolet radiation and electric fields (Rothwell et al., 2022). In contrast, the present study focused exclusively on indirect exposure to PAL, allowing isolation of the chemical effects of plasma-generated species.

Consistent with prior work, we observed a tendency toward very strong susceptibility among Gram-negative species (mean reduction ~6.3 log_10_ vs. ~2.6 log_10_ for Gram-positives). This tendency is commonly attributed to structural differences in the bacterial cell envelope, particularly the thicker peptidoglycan layer of Gram-positive bacteria, which can confer increased resistance to oxidative and nitrosative stress (Mai-Prochnow et al., 2014). However, this distinction was not absolute: *P. aeruginosa* (Gram-negative) exhibited intermediate to strong resilience (~5.3 log_10_ reduction), comparable to the Gram-positive *S. aureus* (~3.2 log_10_ reduction). This highlights that PAL susceptibility is determined by an interplay of cell wall structure and species-specific defense mechanisms, such as robust antioxidant systems or biofilm-like aggregation, rather than Gram status alone (Zhang et al., 2023).

### 4.2 FeTPPS partially inhibits antibacterial activity, highlighting the role of peroxynitrite

The scavenger experiments provide indirect mechanistic insight into the reactive species contributing to PAL-mediated antibacterial activity (Figure 4). The significant attenuation observed with FeTPPS is consistent with a pivotal role for peroxynitrite (ONOO^−^) and its protonated form, peroxynitrous acid (ONOOH). Under the strongly acidic conditions generated by our APC source (pH <3), NO_2_^−^ is chemically unstable and reacts with H_2_O_2_ to form ONOOH. This short-lived species is a potent oxidant known to induce severe nitrosative stress at bacterial membranes (Lukes et al., 2014; Zhou et al., 2018). The fact that FeTPPS, which catalyzes the isomerization of peroxynitrite to nitrate, rescued bacterial viability confirms that this specific secondary species is a key effector of toxicity in our system which has also been reported elsewhere (Ma et al., 2020).

In contrast, scavengers targeting singlet oxygen (^1^O_2_, L-histidine), hypochlorous acid (HOCl, taurine), and hydroxyl radicals (•OH, D-mannitol) did not demonstrate any measurable inhibitory effect when the pH was maintained at 2.8. In the presence of these scavengers, the antibacterial efficacy of PAL was even slightly increased rather than diminished. While such observations may partly be attributed to the inherent variability of biological systems, it is also conceivable that scavenger addition altered the biochemical pathways within PAL, potentially increasing the concentration or availability of other antibacterial species such as peroxynitrite.

Because L-histidine increased pH when used without adjustment, we applied a pH-matched L-histidine condition (pH 2.8 ± 0.2). Under pH-matched conditions no inhibition was observed, indicating that the apparent loss of activity in the unadjusted condition was primarily pH-driven rather than attributable to ^1^O_2_ scavenging. In contrast, some literature attributed antibacterial activity to superoxide (O₂^−^) or other species (Ma et al., 2020; Rothwell et al., 2022). Furthermore, other studies utilizing similar scavengers reported more pronounced inhibitory effects than those observed in our experimental system, e.g., for D-mannitol or L-histidine (Ma et al., 2020; Kang et al., 2022; Xu et al., 2023).

These discrepancies across studies may be due to varying concentrations, plasma-generation parameters, and types of PAL, each potentially favoring distinct biochemical pathways. Future investigations into these complex chemical interactions will be necessary to further clarify the roles of specific short-lived species under diverse PAL generation conditions.

### 4.3 Clinical translation and limitations

This study demonstrates that relatively large and clinically relevant volumes of PAL (50 mL) can be generated within a short time frame (<5 min) using a certified electrosurgical APC plasma source. The ability to adjust power settings and increase power output makes this system more scalable and time-efficient than typical cold plasma generators operating at near body temperature. Furthermore, as the treated liquid functions as a heat sink, excessive temperature buildup can be effectively minimized, ensuring safe handling and stability of the resulting PAL.

Our findings confirm that PAL produced with the APC system exhibits strong antibacterial efficacy against several clinically important pathogens, including *H. pylori* and *E. coli* (MDR). While previous studies have shown PAL efficacy against ESKAPE pathogens (Scholtz et al., 2021), this present study extends these observations to the inactivation of *H. pylori in vitro*. Previous studies investigated the direct plasma treatment of *H. pylori* colonized surfaces and attributed a part of the effect on the UV radiation which can be excluded in this scenario (Sakudo et al., 2018).

Both non-resistant and antibiotic-resistant *E. coli* strains showed comparable susceptibility to PAL, consistent with a mode of action that differs fundamentally from conventional antibiotics. Previous studies have suggested that PAL exposure may alter bacterial stress responses or antibiotic susceptibility profiles (Guo et al., 2018; Vuković et al., 2025); however, such effects were not directly assessed here and require dedicated investigation.

Given the strong pH dependence observed in this study, acidic environments may represent relevant contexts for further investigation of PAL activity. In this regard, the gastric lumen, characterized by low pH, provides a physiologically acidic setting in which PAL-mediated antibacterial effects could be explored experimentally. *H. pylori*, a pathogen adapted to survive within the gastric mucosa and increasingly resistant to standard antibiotic regimens, represents a relevant model organism for such investigations. Chronic *H. pylori* infection is a major risk factor for peptic ulcer disease and gastric cancer (Katelaris et al., 2023; Park et al., 2025).

An important translational question is whether the long exposure times used in some suspension assays are feasible and required in the gastrointestinal tract. Our time-course analysis revealed that *H. pylori* is exceptionally sensitive, showing a >4.5 log_10_ reduction within 15 min. This demonstrates that prolonged exposure (e.g., hours) is not strictly necessary for this pathogen. Accordingly, an endoscopic approach involving targeted irrigation or a transient “flooding” of 10–15 min could be clinically feasible to significantly reduce bacterial load. In refractory scenarios, PAL could be explored as a bacterial load-reducing adjunct prior to standard eradication regimens, but any impact on clinical eradication outcomes remains speculative without *in vivo* evidence.

Beyond the gastrointestinal tract, PAL has been discussed as a potential approach for antibacterial control in other acidic or locally confined environments, including biofilm-associated infections (Mai-Prochnow et al., 2021) and applications in endoscopic or dental settings (Koga-Ito et al., 2023). However, these possibilities require dedicated *in vivo* studies to assess feasibility, safety, and efficacy.

Several limitations should be acknowledged. All experiments were conducted under controlled *in vitro* conditions using a limited panel of bacterial strains and defined exposure times. The behavior of PAL in complex biological environments, including buffering effects, organic load, tissue interactions, and host responses, was not assessed. Moreover, the activity of APC-generated PAL against other microorganisms such as fungi or viruses, as well as its physicochemical stability and safety in living tissues, remains to be determined.

In summary, this study provides an *in vitro* characterization of antibacterial activity and underlying chemical dependencies of APC-generated PAL. While the results support further investigation of this approach, additional preclinical and clinical studies are required to assess safety, efficacy, and practical applicability under physiologically relevant conditions.

## Data Availability

The original contributions presented in the study are included in the article/supplementary material, further inquiries can be directed to the corresponding author.

## References

[ref1] Adamovich I. Agarwal S. Ahedo E. Alves L. L. Baalrud S. Babaeva N. . (2022). The 2022 plasma roadmap: low temperature plasma science and technology. J. Phys. D: Appl. Phys. 55:373001. doi: 10.1088/1361-6463/Ac5e1c

[ref2] Amini M. R. Sheikh Hosseini M. Fatollah S. Mirpour S. Ghoranneviss M. Larijani B. . (2020). Beneficial effects of cold atmospheric plasma on inflammatory phase of diabetic foot ulcers; a randomized clinical trial. J. Diabetes Metab. Disord. 19, 895–905. doi: 10.1007/S40200-020-00577-2, 33520811 PMC7843664

[ref3] Argueta E. A. Alsamman M. A. Moss S. F. D’Agata E. M. C. (2021). Impact of antimicrobial resistance rates on eradication of *Helicobacter pylori* in a US population. Gastroenterology 160, 2181–2183.e1. doi: 10.1053/J.Gastro.2021.02.014, 33577874 PMC9115583

[ref4] Bauer G. Sersenová D. Graves D. B. Machala Z. (2019). Cold atmospheric plasma and plasma-activated medium trigger RONS-based tumor cell apoptosis. Sci. Rep. 9:14210. doi: 10.1038/S41598-019-50291-0, 31578342 PMC6775051

[ref5] Bekeschus S. von Woedtke T. Emmert S. Schmidt A. (2021). Medical gas plasma-stimulated wound healing: evidence and mechanisms. Redox Biol. 46:102116. doi: 10.1016/J.Redox.2021.102116, 34474394 PMC8408623

[ref6] Bruggeman P. J. Kushner M. J. Locke B. R. Gardeniers J. G. E. Graham W. G. Graves D. B. . (2016). Plasma–liquid interactions: a review and roadmap. Plasma Sources Sci. Technol. 25:53002. doi: 10.1088/0963-0252/25/5/053002

[ref7] Clemen R. Singer D. Skowski H. Bekeschus S. (2023). Argon humidification exacerbates antimicrobial and anti-MRSA kINPen plasma activity. Life 13:257. doi: 10.3390/Life13020257, 36836614 PMC9968137

[ref8] Das S. Gajula V. P. Mohapatra S. Singh G. Kar S. (2022). Role of cold atmospheric plasma in microbial inactivation and the factors affecting its efficacy. Health Sci. Rev. 4:100037. doi: 10.1016/J.Hsr.2022.100037

[ref9] Gohain R. B. Biswas S. (2025). Impact of applied voltage, air gap, and ground arrangement on discharge power and dielectric capacitance in a volume DBD plasma. Phys. Scr. 100:25604. doi: 10.1088/1402-4896/Ada3f3

[ref10] Grund K. E. Straub T. Farin G. (1999). New haemostatic techniques: argon plasma coagulation. Baillieres Best Pract. Res. Clin. Gastroenterol. 13, 67–84. doi: 10.1053/Bega.1999.0009, 11030635

[ref11] Grund K. E. Zindel C. Farin G. (1997). Argonplasmakoagulation In Der Flexiblen Endoskopie. Bewertung Eines Neuen Therapeutischen Verfahrens Nach 1606 Anwendungen. Dtsch. Med. Wochenschr. 122, 432–438. doi: 10.1055/S-2008-1047634, 9138921

[ref12] Guo L. Xu R. Zhao Y. Liu D. Liu Z. Wang X. . (2018). Gas plasma pre-treatment increases antibiotic sensitivity and persister eradication in methicillin-resistant *Staphylococcus aureus*. Front. Microbiol. 9:537. doi: 10.3389/Fmicb.2018.00537, 29628915 PMC5876240

[ref13] Hooi J. K. Y. Lai W. Y. Ng W. K. Suen M. M. Y. Underwood F. E. Tanyingoh D. . (2017). Global prevalence of *Helicobacter pylori* infection: systematic review and meta-analysis. Gastroenterology 153, 420–429. doi: 10.1053/J.Gastro.2017.04.022, 28456631

[ref14] Ikawa S. Kitano K. Hamaguchi S. (2010). Effects of pH on bacterial inactivation in aqueous solutions due to low-temperature atmospheric pressure plasma application. Plasma Process. Polym. 7, 33–42. doi: 10.1002/Ppap.200900090

[ref15] Kang T. Yim D. Baek K. H. Lee Y. E. Kim H.-J. Jo C. (2022). The inactivation efficacy of plasma-activated acetic acid against *Salmonella* Typhimurium cells and biofilm. J. Appl. Microbiol. 133, 3007–3019. doi: 10.1111/Jam.15757, 35916587

[ref16] Katelaris P. Hunt R. Bazzoli F. Cohen H. Fock K. M. Gemilyan M. . (2023). *Helicobacter pylori* world gastroenterology organization global guideline. J. Clin. Gastroenterol. 57, 111–126. doi: 10.1097/Mcg.0000000000001719, 36598803

[ref17] Kaushik N. K. Ghimire B. Li Y. Adhikari M. Veerana M. Kaushik N. . (2018). Biological and medical applications of plasma-activated media, water and solutions. Biol. Chem. 400, 39–62. doi: 10.1515/Hsz-2018-0226, 30044757

[ref18] Kim S. Kim C.-H. (2021). Applications of plasma-activated liquid in the medical field. Biomedicine 9:1700. doi: 10.3390/Biomedicines9111700, 34829929 PMC8615748

[ref19] Koga-Ito C. Y. Kostov K. G. Miranda F. S. Milhan N. V. Azevedo Neto N. F. Nascimento F. . (2023). Cold atmospheric plasma as a therapeutic tool in medicine and dentistry. Plasma Chem. Plasma Process. 44, 1393–1429. doi: 10.1007/S11090-023-10380-5

[ref20] Laroussi M. Bekeschus S. Keidar M. Bogaerts A. Fridman A. Lu X. . (2022). Low-temperature plasma for biology, hygiene, and medicine: perspective and roadmap. IEEE Trans. Radiat. Plasma Med. Sci. 6, 127–157. doi: 10.1109/Trpms.2021.3135118

[ref21] Liou J.-M. Malfertheiner P. Lee Y.-C. Sheu B.-S. Sugano K. Cheng H.-C. . (2020). Screening and eradication of *Helicobacter pylori* for gastric cancer prevention: the Taipei global consensus. Gut 69, 2093–2112. doi: 10.1136/Gutjnl-2020-322368, 33004546

[ref22] Liu F. Sun P. Bai N. Tian Y. Zhou H. Wei S. . (2010). Inactivation of bacteria in an aqueous environment by a direct-current, cold-atmospheric-pressure air plasma microjet. Plasma Process. Polym. 7, 231–236. doi: 10.1002/Ppap.200900070

[ref23] Lukes P. Dolezalova E. Sisrova I. Clupek M. (2014). Aqueous-phase chemistry and bactericidal effects from an air discharge plasma in contact with water: evidence for the formation of peroxynitrite through a pseudo-second-order post-discharge reaction of H_2_O_2_ and HNO_2_. Plasma Sources Sci. Technol. 23:15019. doi: 10.1088/0963-0252/23/1/015019

[ref24] Ma M. Zhang Y. Lv Y. Sun F. (2020). The key reactive species in the bactericidal process of plasma activated water. J. Phys. D: Appl. Phys. 53:185207. doi: 10.1088/1361-6463/Ab703a

[ref25] Mai-Prochnow A. Murphy A. B. Mclean K. M. Kong M. G. Ostrikov K. K. (2014). Atmospheric pressure plasmas: infection control and bacterial responses. Int. J. Antimicrob. Agents 43, 508–517. doi: 10.1016/J.Ijantimicag.2014.01.025 24637224

[ref26] Mai-Prochnow A. Zhou R. Zhang T. Ostrikov K. K. Mugunthan S. Rice S. A. . (2021). Interactions of plasma-activated water with biofilms: inactivation, dispersal effects and mechanisms of action. npj Biofilms Microbiomes 7:11. doi: 10.1038/S41522-020-00180-6, 33504802 PMC7841176

[ref27] Malfertheiner P. Megraud F. O'morain C. A. Atherton J. Axon A. T. R. Bazzoli F. . (2012). Management of *Helicobacter pylori* infection—the Maastricht IV/ Florence consensus report. Gut 61, 646–664. doi: 10.1136/Gutjnl-2012-302084, 22491499

[ref28] Martinet A. Miebach L. Heisterberg L. Neugebauer A. Enderle M. D. Bekeschus S. (2025). Reactive species production and colon cancer cytotoxicity of an electrosurgical cold argon plasma device. Plasma Process. Polym. 22:e2400240. doi: 10.1002/Ppap.202400240

[ref29] Marzi J. Stope M. B. Henes M. Koch A. Wenzel T. Holl M. . (2022). Noninvasive physical plasma as innovative and tissue-preserving therapy for women positive for cervical intraepithelial neoplasia. Cancers 14:1933. doi: 10.3390/Cancers14081933, 35454839 PMC9027888

[ref30] Milhan N. V. M. Chiappim W. Da Sampaio A. G. Da Vegian M. R. C. Pessoa R. S. Koga-Ito C. Y. (2022). Applications of plasma-activated water in dentistry: a review. Int. J. Mol. Sci. 23:4131. doi: 10.3390/Ijms23084131 35456947 PMC9029124

[ref31] Montalbetti R. Machala Z. Gherardi M. Laurita R. (2025). Production and chemical composition of plasma activated water: a systematic review and meta-analysis. Plasma Process. Polym. 22:2400249. doi: 10.1002/Ppap.202400249

[ref32] Oehmigen K. Hähnel M. Brandenburg R. Wilke C. Weltmann K.-D. von Woedtke T. (2010). The role of acidification for antimicrobial activity of atmospheric pressure plasma in liquids. Plasma Process. Polym. 7, 250–257. doi: 10.1002/Ppap.200900077

[ref33] Park J. Y. Georges D. Alberts C. J. Bray F. Clifford G. Baussano I. (2025). Global lifetime estimates of expected and preventable gastric cancers across 185 countries. Nat. Med. 31, 3020–3027. doi: 10.1038/S41591-025-03793-6, 40624406 PMC12443596

[ref34] Puač N. Škoro N. (2025). Plasma–liquid interaction for agriculture—a focused review. Plasma Process. Polym. 22:2400208. doi: 10.1002/Ppap.202400208

[ref35] Reuter S. von Woedtke T. Weltmann K.-D. (2018). The kINPen—a review on physics and chemistry of the atmospheric pressure plasma jet and its applications. J. Phys. D: Appl. Phys. 51:233001. doi: 10.1088/1361-6463/Aab3ad

[ref36] Rothwell J. G. Alam D. Carter D. A. Soltani B. Mcconchie R. Zhou R. . (2022). The antimicrobial efficacy of plasma-activated water against *Listeria* and *E. coli* is modulated by reactor design and water composition. J. Appl. Microbiol. 132, 2490–2500. doi: 10.1111/Jam.15429, 34957649

[ref37] Rotondo P. R. Aceto D. Ambrico M. Stellacci A. M. Faretra F. De Miccolis Angelini R. M. . (2025). Physicochemical properties of plasma-activated water and associated antimicrobial activity against Fungi and Bacteria. Sci. Rep. 15:5536. doi: 10.1038/S41598-025-88369-7, 39953074 PMC11828987

[ref38] Sakudo A. Miyagi H. Horikawa T. Yamashiro R. Misawa T. (2018). Treatment of *Helicobacter pylori* with dielectric barrier discharge plasma causes UV induced damage to genomic DNA leading to cell death. Chemosphere 200, 366–372. doi: 10.1016/J.Chemosphere.2018.02.115 29494918

[ref39] Salam M. A. Al-Amin M. Y. Salam M. T. Pawar J. S. Akhter N. Rabaan A. A. . (2023). Antimicrobial resistance: a growing serious threat for global public health. Healthcare 11:1946. doi: 10.3390/Healthcare11131946, 37444780 PMC10340576

[ref40] Scholtz V. Vaňková E. Kašparová P. Premanath R. Karunasagar I. Julák J. (2021). Non-thermal plasma treatment of ESKAPE pathogens: a review. Front. Microbiol. 12:737635. doi: 10.3389/Fmicb.2021.737635, 34712211 PMC8546340

[ref41] Talukdar P. Gohain R. B. Bharadwaj P. Thakur D. Biswas S. (2025). Inactivation of *Candida albicans*, staphylococcus aureus and multidrug-resistant *Escherichia coli* with dielectric barrier discharged cold atmospheric plasma: a comparative study with antimicrobial drugs. J. Med. Microbiol. 74:1965. doi: 10.1099/Jmm.0.001965 39879135

[ref42] Traylor M. J. Pavlovich M. J. Karim S. Hait P. Sakiyama Y. Clark D. S. . (2011). Long-term antibacterial efficacy of air plasma-activated water. Plasma Sources Sci. Technol. 44:472001. doi: 10.1088/0022-3727/44/47/472001

[ref43] Veronico V. Favia P. Fracassi F. Gristina R. Sardella E. (2021). Validation of colorimetric assays for hydrogen peroxide, nitrate and nitrite ions in complex plasma-treated water solutions. Plasma Process. Polym. 18:2100062. doi: 10.1002/Ppap.202100062

[ref44] von Woedtke T. Bekeschus S. Weltmann K.-D. Wende K. (2025). Plasma-treated liquids for medicine: a narrative review on state and perspectives. Plasma Process. Polym. 22:2400255. doi: 10.1002/Ppap.202400255

[ref45] Vuković D. Miletić M. Toljić B. Milojević N. Jovanović O. Kuzmanović Pfićer J. . (2025). Plasma-activated water against Carbapenem-resistant *Klebsiella pneumoniae* and vancomycin-resistant *Enterococcus faecalis*. Pathogens 14:410. doi: 10.3390/Pathogens14050410, 40430731 PMC12114337

[ref46] Wang Q. Salvi D. (2021). Evaluation of plasma-activated water (paw) as a novel disinfectant: effectiveness on *Escherichia coli* and *Listeria innocua*, physicochemical properties, and storage stability. LWT 149:111847. doi: 10.1016/J.Lwt.2021.111847

[ref47] Wang L. Xia C. Guo Y. Yang C. Cheng C. Zhao J. . (2020). Bactericidal efficacy of cold atmospheric plasma treatment against multidrug-resistant *Pseudomonas aeruginosa*. Future Microbiol. 15, 115–125. doi: 10.2217/Fmb-2019-0265, 31989838

[ref48] Weiss M. Arnholdt M. Hißnauer A. Fischer I. Schönfisch B. Andress J. . (2023). Tissue-preserving treatment with non-invasive physical plasma of cervical intraepithelial neoplasia-a prospective controlled clinical trial. Front. Med. 10:1242732. doi: 10.3389/Fmed.2023.1242732, 37654659 PMC10465690

[ref49] Weiss M. Utz R. Ackermann M. Taran F.-A. Krämer B. Hahn M. . (2019). Characterization of a non-thermally operated electrosurgical argon plasma source by electron spin resonance spectroscopy. Plasma Process. Polym. 16:1800150. doi: 10.1002/Ppap.201800150

[ref50] Weltmann K. D. Kindel E. von Woedtke T. Hähnel M. Stieber M. Brandenburg R. (2010). Atmospheric-pressure plasma sources: prospective tools for plasma medicine. Pure Appl. Chem. 82, 1223–1237. doi: 10.1351/Pac-Con-09-10-35

[ref51] Xu H. Fang C. Shao C. Li L. Huang Q. (2022). Study of the synergistic effect of singlet oxygen with other plasma-generated ROS in fungi inactivation during water disinfection. Sci. Total Environ. 838:156576. doi: 10.1016/J.Scitotenv.2022.156576, 35688233

[ref52] Xu H. Liu C. Huang Q. (2023). Enhance the inactivation of fungi by the sequential use of cold atmospheric plasma and plasma-activated water: synergistic effect and mechanism study. Chem. Eng. J. 452:139596. doi: 10.1016/J.Cej.2022.139596

[ref53] Zenker M. (2008). Argon plasma coagulation. GMS Krankenhaushyg. Interdiszip. 3:Doc15. Available online at: https://pmc.ncbi.nlm.nih.gov/articles/PMC2831517/PMC2831517 20204117

[ref54] Zhang H. Zhang C. Han Q. (2023). Mechanisms of bacterial inhibition and tolerance around cold atmospheric plasma. Appl. Microbiol. Biotechnol. 107, 5301–5316. doi: 10.1007/S00253-023-12618-W, 37421472 PMC10390405

[ref55] Zhao Y.-M. Ojha S. Burgess C. M. Sun D.-W. Tiwari B. K. (2020). Inactivation efficacy and mechanisms of plasma activated water on bacteria in planktonic state. J. Appl. Microbiol. 129, 1248–1260. doi: 10.1111/Jam.14677, 32358824

[ref56] Zhao Y.-M. Ojha S. Burgess C. M. Sun D.-W. Tiwari B. K. (2021). Inactivation efficacy of plasma-activated water: influence of plasma treatment time, exposure time and bacterial species. Int. J. Food Sci. Technol. 56, 721–732. doi: 10.1111/Ijfs.14708

[ref57] Zhou R. Zhou R. Prasad K. Fang Z. Speight R. Bazaka K. . (2018). Cold atmospheric plasma activated water as a prospective disinfectant: the crucial role of peroxynitrite. Green Chem. 20, 5276–5284. doi: 10.1039/C8gc02800a

